# The hypocretin/orexin antagonist almorexant promotes sleep without impairment of performance in rats

**DOI:** 10.3389/fnins.2014.00003

**Published:** 2014-01-31

**Authors:** Stephen R. Morairty, Alan J. Wilk, Webster U. Lincoln, Thomas C. Neylan, Thomas S. Kilduff

**Affiliations:** ^1^SRI International, Center for Neuroscience, Biosciences DivisionMenlo Park, CA, USA; ^2^Department of Psychiatry, SF VA Medical Center/NCIRE/University of CaliforniaSan Francisco, CA, USA

**Keywords:** hypocretins/orexins, cognitive impairment, memory impairment, hypnotics, water maze, spatial reference memory, spatial working memory, EEG

## Abstract

The hypocretin receptor (HcrtR) antagonist almorexant (ALM) has potent hypnotic actions but little is known about neurocognitive performance in the presence of ALM. HcrtR antagonists are hypothesized to induce sleep by disfacilitation of wake-promoting systems whereas GABA_A_ receptor modulators such as zolpidem (ZOL) induce sleep through general inhibition of neural activity. To test the hypothesis that less functional impairment results from HcrtR antagonist-induced sleep, we evaluated the performance of rats in the Morris Water Maze in the presence of ALM vs. ZOL. Performance in spatial reference memory (SRM) and spatial working memory (SWM) tasks were assessed during the dark period after equipotent sleep-promoting doses (100 mg/kg, po) following undisturbed and sleep deprivation (SD) conditions. ALM-treated rats were indistinguishable from vehicle (VEH)-treated rats for all SRM performance measures (distance traveled, latency to enter, time within, and number of entries into, the target quadrant) after both the undisturbed and 6 h SD conditions. In contrast, rats administered ZOL showed impairments in all parameters measured compared to VEH or ALM in the undisturbed conditions. Following SD, ZOL-treated rats also showed impairments in all measures. ALM-treated rats were similar to VEH-treated rats for all SWM measures (velocity, time to locate the platform and success rate at finding the platform within 60 s) after both the undisturbed and SD conditions. In contrast, ZOL-treated rats showed impairments in velocity and in the time to locate the platform. Importantly, ZOL rats only completed the task 23–50% of the time while ALM and VEH rats completed the task 79–100% of the time. Thus, following equipotent sleep-promoting doses, ZOL impaired rats in both memory tasks while ALM rats performed at levels comparable to VEH rats. These results are consistent with the hypothesis that less impairment results from HcrtR antagonism than from GABA_A_-induced inhibition.

## Introduction

Insomnia is a highly prevalent condition affecting 10–30% of the general population; (NIH, 2005; Roth, 2007; Mai and Buysse, 2008). Sleep loss and sleep disruption can lead to a degradation of neurocognitive performance as assessed by objective and subjective measures (Wesensten et al., 1999; Belenky et al., 2003; Lamond et al., 2007). Prescription sleep medications are often used to treat insomnia and obtain desired amounts of sleep. Presently, nonbenzodiazepine, positive allosteric modulators of the GABA_A_ receptor such as zolpidem (ZOL) are the most widely prescribed hypnotic medications. Although known to induce sleep, these compounds have been shown to significantly impair psychomotor and memory functions in rodents (Huang et al., 2010; Uslaner et al., 2013; Zanin et al., 2013), non-human primates (Makaron et al., 2013; Soto et al., 2013; Uslaner et al., 2013) and humans (Balkin et al., 1992; Wesensten et al., 1996, 2005; Mattila et al., 1998; Mintzer and Griffiths, 1999; Verster et al., 2002; Storm et al., 2007; Otmani et al., 2008; Gunja, 2013). Such impairment can be particularly troubling when there is an urgent need for highly functional performance in the presence of drug such as with first responders, military personnel, and caregivers. Further, complex behaviors during the sleep period (e.g., eating, cooking, driving, conversations, sex) have been associated with these medications (Dolder and Nelson, 2008). Therefore, more effective hypnotics are needed that facilitate sleep that is easily reversible in the event of an unexpected awakening that demands unimpaired cognitive and psychomotor performance.

Recently, antagonism of the hypocretin (Hcrt; also called orexin) receptors has been identified as a target mechanism for the next generation of sleep medications (Brisbare-Roch et al., 2007; Dugovic et al., 2009; Whitman et al., 2009; Hoever et al., 2010, 2012a,b; Coleman et al., 2012; Herring et al., 2012; Winrow et al., 2012; Betschart et al., 2013). The Hcrt system is well known to play an important role in the maintenance of wakefulness (de Lecea, 2012; Inutsuka and Yamanaka, 2013; Mieda and Sakurai, 2013; Saper, 2013). Hcrt fibers project throughout the central nervous system (CNS), with particularly dense projections and receptor expression found in arousal centers including the locus coeruleus, the tuberomammilary nucleus, dorsal raphe nuclei, laterodorsal tegmentum, pedunculopontine tegmentum, and the basal forebrain (Peyron et al., 1998; Marcus et al., 2001). The excitatory effects of the Hcrt peptides on these arousal centers is hypothesized to stabilize and maintain wakefulness. Therefore, blockade of the Hcrt system should disfacilitate these arousal centers, creating conditions that are permissive for sleep to occur.

The current study tests the hypothesis that the dual Hcrt receptor antagonist almorexant (ALM) produces less functional impairment than ZOL. The rationale that underlies this hypothesis is that ZOL causes a general inhibition of neural activity whereas ALM specifically disfacilitates wake-promoting systems. We tested this hypothesis using tests of spatial reference memory (SRM) and spatial working memory (SWM) in the Morris Water Maze. Although the concentrations of ALM and ZOL administered prior to these tests were equipotent in hypnotic efficacy, the performance of rats treated with ALM were superior to that of rats treated with ZOL.

## Materials and methods

### Animals

One hundred fifty three male Sprague Dawley rats (300 g at time of purchase; Charles River, Wilmington, MA) were distributed among the 12 groups as described in Table 1. All animals were individually housed in temperature-controlled recording chambers (22 ± 2°C, 50 ± 25% relative humidity) under a 12:12 light/dark cycle with food and water available *ad libitum*. All experimental procedures were approved by SRI International's Institutional Animal Care and Use Committee and were in accordance with National Institute of Health (NIH) guidelines.

**Table 1:** **The number of rats tested for each of the 12 experimental groups**.

**Test**	**No SD**			**6 h SD**		
	**VEH**	**ALM**	**ZOL**	**VEH**	**ALM**	**ZOL**
Reference memory	14	13	17	16	16	8
Working memory	11	12	12	12	11	11

### Surgical procedures

Rats were instrumented with sterile telemetry transmitters (F40-EET, Data Sciences Inc., St Paul, MN) as previously described (Morairty et al., 2008, 2012; Revel et al., 2012, 2013). Briefly, under isoflurane anesthesia, transmitters were placed intraperitoneally and biopotential leads were routed subcutaneously to the head and neck. Holes were drilled into the skull at 1.5 mm anterior to bregma and 1.5 mm lateral to midline, and 6 mm posterior to bregma and 4 mm lateral to midline on the right hemisphere. Two biopotential leads used as EEG electrodes were inserted into the holes and affixed to the skull with dental acrylic. Two biopotential leads used as EMG electrodes were positioned bilaterally through the nuchal muscles.

### Identification of sleep/wake states

After at least 3 weeks post-surgical recovery, EEG, and EMG were recorded via telemetry using DQ ART 4.1 software (Data Sciences Inc., St Paul, MN). Following completion of data collection, the EEG, and EMG recordings were scored in 10 s epochs as waking (W), rapid eye movement sleep (REM), or non-rapid eye movement sleep (NREM) by expert scorers blinded to the treatments using NeuroScore software (Data Sciences Inc., St Paul, MN). Sleep latency was defined as the first 60 s of continuous sleep following drug administration. Recordings were started at Zeitgeber time (ZT) 12 (lights off) and continued until animals performed the water maze tests.

### Sleep deprivation procedures

Animals were sleep deprived (SD) from ZT12-18 by progressive manual stimulation concurrent with EEG and EMG recording. The rats were continuously observed and, when they appeared to attempt to sleep, progressive interventions were employed to keep them awake: removal of cage tops, tapping on cages, placement of brushes inside the cage, or stroking of vibrissae or fur with an artist's brush.

### Drugs

Almorexant (ALM; ACT-078573), was synthesized at SRI International (Menlo Park, CA. USA) according to the patent literature. Zolpidem (ZOL) was a gift from Actelion Pharmaceuticals Ltd. For the SWM task, rats were dosed with ALM (100 mg/kg, p.o.), ZOL (100 mg/kg, p.o.) or vehicle (VEH; 1.25% hydroxypropyl methyl cellulose, 0.1% dioctyl sodium sulfosuccinate, and 0.25% methyl cellulose in water) at ZT18 and left undisturbed until time to perform memory tasks (see below). For the SRM task, most rats were also administered ALM, ZOL, and VEH p.o. at the concentrations above. However, one cohort of rats was administered drugs i.p. For these rats, ALM was administered at 100 mg/kg (*N* = 6), ZOL at 30 mg/kg (*N* = 8) and VEH (*N* = 7). ZOL is approximately 3X more potent i.p. than p.o.(Vanover et al., 1999) while ALM is equipotent through both routes of administration. Analysis of the sleep/wake data confirmed the equipotent effects of both drugs through both routes of administration at the concentrations tested.

### Water maze

**Figure 1:**
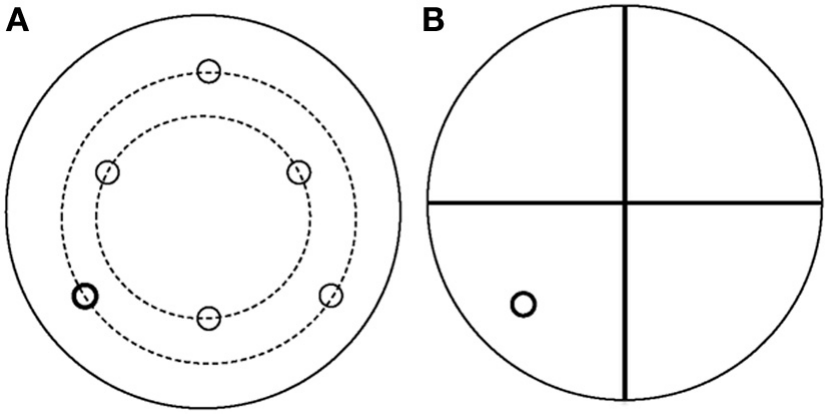
**Schematic of the water maze apparatus used for both spatial reference and spatial working memory tasks. (A)** Schematic of the platform locations. **(B)** Example of quadrant orientations used for analysis used for the platform indicated in bold. Quadrant locations were always oriented so that the platform was central within a quadrant.

All water maze (WM) tasks occurred in a pool 68″ in diameter and 25″ in depth, containing water at 24 ± 2°C made opaque by the addition of non-toxic, water soluble black paint and milk powder. Since all tests took place during the dark period, distinctive spatial cues were made of small “rice” lights colored blue, yellow, and green. Patterns of lights in distinct shapes (circle, square, diamond, “T” shape) were clearly visible from within the pool. Preliminary studies determined the minimum number of lights that were needed for learning to occur. A 10 cm diameter platform was submerged approximately 1 cm below the surface of the water in one of 6 locations (Figure 1). The platform location determined the orientation of the 4 quadrants used for analysis. Both WM tasks were similar to previous reports (Wenk, 2004; Ward et al., 2009).

### Test of spatial reference memory

The acquisition phase occurred in one session consisting of 12–15 consecutive trials with a 60 s inter-trial interval. For each trial, rats were placed in the WM facing the wall in one of three quadrants that did not contain the hidden platform. The location of the hidden platform remained constant across all trials. Rats were given 60 s to locate the platform. If the rats did not locate the platform within this period, they were guided to the platform location. When the rats reached the platform, they were allowed to remain on the platform for approximately 15 s before being placed in a dry holding cage for the next 60 s. This training sequence continued until the rats learned the task, typically 12–15 trials.

On the following day, rats were dosed with ALM, ZOL or VEH at ZT18 and a retention probe trial was performed 90 min later in which the rats were returned to the WM but the platform had been removed. A total of 40 rats were subjected to SD for 6 h prior to drug administration, and 42 were left undisturbed during this period (Table 1). Rats were started in the quadrant opposite the target quadrant and allowed to swim for 30 s. All trials were recorded by video camera and analyzed with Ethovision XT software (Noldus, Leesburg, VA). Test measures for the retention probe were time spent in target quadrant, latency to target quadrant, frequency of entrance into target quadrant, and total distance traveled. Swim speed was calculated to control for nonspecific effects.

### Test of spatial working memory

The SWM task consisted of 6 pairs of trials, one for each platform location (Figure 1A). In the first trial, a cued platform marked with a flag was placed in one of 6 positions in the WM. Rats were released facing the wall from one of the 3 quadrants not containing the platform and were allowed 120 s to locate the cued platform before the researcher guided the rats to the platform. This procedure provided all rats the opportunity to learn the platform location even if they did not find it on their own. After 15 s on the platform, the rats were removed from the WM and placed in a holding cage. The flag was then removed but the platform remained in the same location as in the first trial. Following a delay of 1, 5, or 10 min in the holding cage, the rats were placed back in the WM into one of the 2 quadrants that did not contain the platform and was not the starting quadrant during the first trial. Once the rats found the platform, they were removed after approximately 5 s and placed back in a holding cage for 10 min before a new pair of trials with a novel platform location was given. The order of delays was counterbalanced so that each rat was tested twice at 1, 5, or 10 min delays between the cued and hidden platforms. All trials were recorded by video camera and analyzed with Ethovision XT software (Noldus, Leesburg, VA). Test measures were time to locate the platform and the swim velocity during all tests.

### Statistical analysis

Statistical analyses were performed using SigmaPlot 12.3 (Systat Software Inc., San Jose, CA). Sleep/wake data (W, NREM, and REM time) were analyzed in 30 min bins and compared between drug groups using Two-Way mixed-model ANOVA on factors “drug group” (between subjects) and “time” (within subjects). SRM performance parameters (latency, duration and frequency in target quadrant, total distance traveled) were analyzed using a One-Way ANOVA. SWM performance measures (velocity, time to platform, percent found) by delay time were analyzed using Two-Way mixed-model ANOVA on factors “drug group” (between subjects) and “time” (within subjects). Significance levels were set at α = 0.05. When ANOVA indicated significance, Bonferroni *t*-tests were used for *post hoc* analyses.

## Results

**Figure 2:**
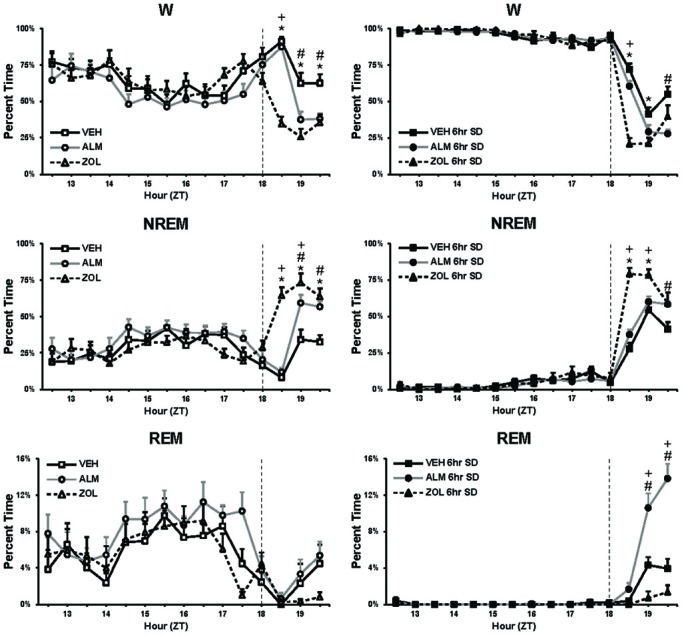
**Percent time spent in W, NREM, and REM during baseline (left panels) and during 6 h SD (right panels)**. The vertical line in each panel at ZT18 depicts the time of drug administration. At the end of the recording time displayed in these panels, rats were tested in the water maze. Note that, for the 60 min prior to testing (ZT19.5), the ALM and ZOL groups slept similar amounts. ^*^, ZOL different from VEH; +, ZOL different ALM; #, ALM different from VEH; *p* < 0.05.

Drug concentrations were chosen to be equipotent at sleep promotion based on our previous experience (Morairty et al., 2012). Although ZOL produced a more rapid onset to sleep under both SD and undisturbed conditions (No SD: ZOL = 6.6 min, VEH = 32.2 min, ALM = 25.4 min; SD: ZOL = 5.9 min, VEH = 20.0 min, ALM = 15.5 min), ALM- and ZOL-treated rats slept equivalent amounts during the last hour before the WM test (Figure 2; No SD: ZOL = 69.4%, ALM = 62.3%, VEH = 37.6%; SD: ZOL = 69.6%, ALM = 71.5%, VEH = 52.0%).

### Test of spatial reference memory

**Figure 3:**
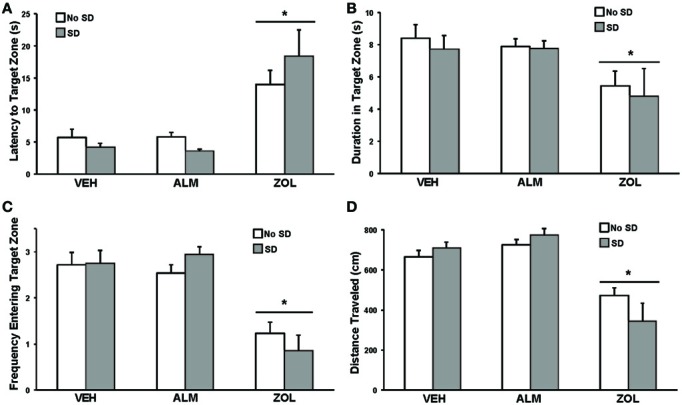
**Measures of performance in the spatial reference memory task**. For all measures, ZOL-treated rats performed poorly compared to VEH- and ALM-treated rats. For all measures, the ALM-treated rats were indistinguishable from the VEH-treated rats. **(A)** Latency to the target zone. **(B)** Duration in the target zone. **(C)** Frequency entering the target zone. **(D)** Total distance traveled. For all measures, ANOVA revealed an effect of drug condition without an effect of SD. ^*^, *p* < 0.05.

For all performance measures analyzed, rats treated with ZOL showed significant impairments while ALM- and VEH-treated rats were indistinguishable (Figure 3). Following ZOL, the latency to the target zone increased (No SD: ZOL = 14.1 s, VEH = 5.7 s, ALM = 5.8 s; SD: ZOL = 18.4 s, VEH = 4.2 s, ALM = 3.6 s) and the duration in the target zone (No SD: ZOL = 5.5 s, VEH = 8.4 s, ALM = 7.9 s; SD: ZOL = 4.8 s, VEH = 7.7 s, ALM = 7.8 s), frequency entering the target zone (No SD: ZOL = 1.2, VEH = 2.7, ALM = 2.5; SD: ZOL = 0.9, VEH = 2.8, ALM = 2.9) and the distance traveled (No SD: ZOL = 472 cm, VEH = 666 cm, ALM = 725 cm; SD: ZOL = 343 cm, VEH = 709 cm, ALM = 775 cm) all decreased compared to VEH and ALM-treated rats. ALM-treated rats did not differ from VEH-treated rats on any of these four measures. Performance in the SRM task was not significantly affected by 6 h SD for any measure within any group.

**Figure 4:**
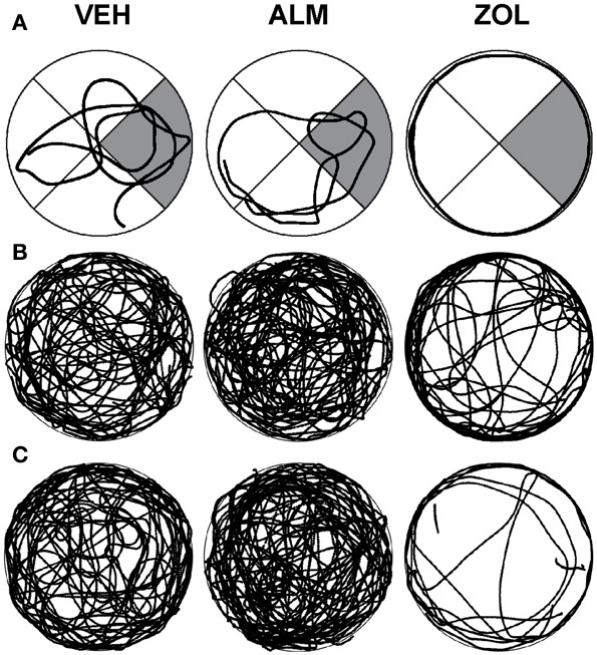
**Swim patterns during the spatial reference memory probe trials following VEH (left columns), ALM (center columns) and ZOL (right columns). (A)** Examples of individual rats. The target quadrant is highlighted in gray. **(B)** Traces for all rats in the undisturbed condition. **(C)** Traces for all rats in the 6 h SD condition. Note that the searching pattern for VEH and ALM are similar while the pattern following ZOL remains primarily around the perimeter of the maze.

Swim patterns in the WM were different for ZOL-treated rats compared to VEH- and ALM-treated rats (Figure 4). Both VEH and ALM rats repeatedly swam across the WM and typically swam through the area where the hidden platform was present on the previous day (Figure 4A). In contrast, ZOL-treated rats primarily swam around the perimeter of the WM, a pattern typical of a rat during its first exposure to the WM.

### Test of spatial working memory

**Figure 5:**
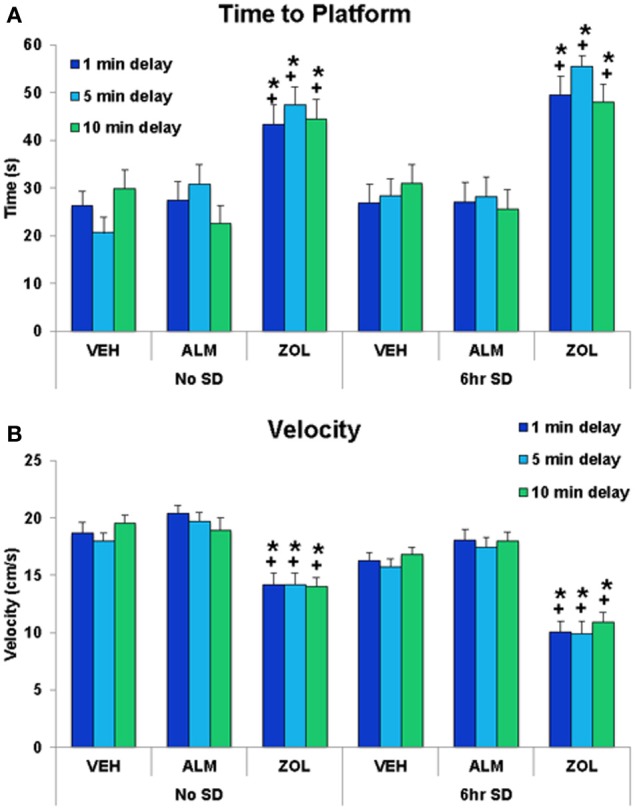
**The time to platform and the velocity swam during the spatial working memory task. (A)** ZOL-treated rats found the platform significantly slower than VEH- or ALM-treated rats for all three delays following either undisturbed or SD conditions. The ALM-treated rats were not significantly different from VEH-treated rats for any condition. **(B)** ZOL-treated rats swam more slowly than either VEH- or ALM-treated rats. ^*^, different from VEH; +, different from ALM; *p* < 0.05.

**Figure 6:**
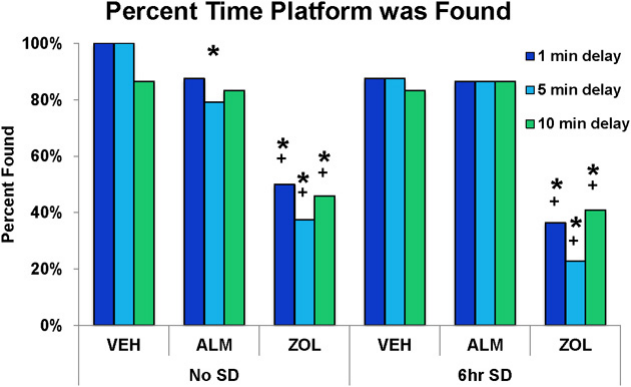
**Success rate in locating the platform during the test trials in the spatial working memory task**. ZOL-treated rats found the platform significantly fewer times compared to VEH- or ALM-treated rats for all three delays and following both the undisturbed and SD conditions. In each trial, an individual rat either found or didn't find the platform; thus, there is no variation to represent as error bars in the graphs. ^*^, different from VEH; +, different from ALM; *p* < 0.05.

ZOL-treated rats performed poorly in the SWM task compared to either VEH- or ALM-treated rats (Figures 5, 6). ZOL-treated rats took longer to find the platform (No SD: ZOL = 43.4–47.3 s, VEH = 20.6–30.0 s, ALM = 22.5–30.7 s; SD: ZOL = 48.0–55.5 s, VEH = 26.9–31.0 s, ALM = 25.6–28.2 s) and swam more slowly (No SD: ZOL = 14.0–14.2 cm/s, VEH = 18.0–19.6 cm/s, ALM = 18.9–20.4 cm/s; SD: ZOL = 9.9–10.9 cm/s, VEH = 15.7–16.8 cm/s, ALM = 17.5–18.1 cm/s) than the VEH or ALM rats (Figure 5). These measures were not affected by increasing the delay from 1 to 5 min or 10 min for any of the 6 groups of rats.

**Figure 7:**
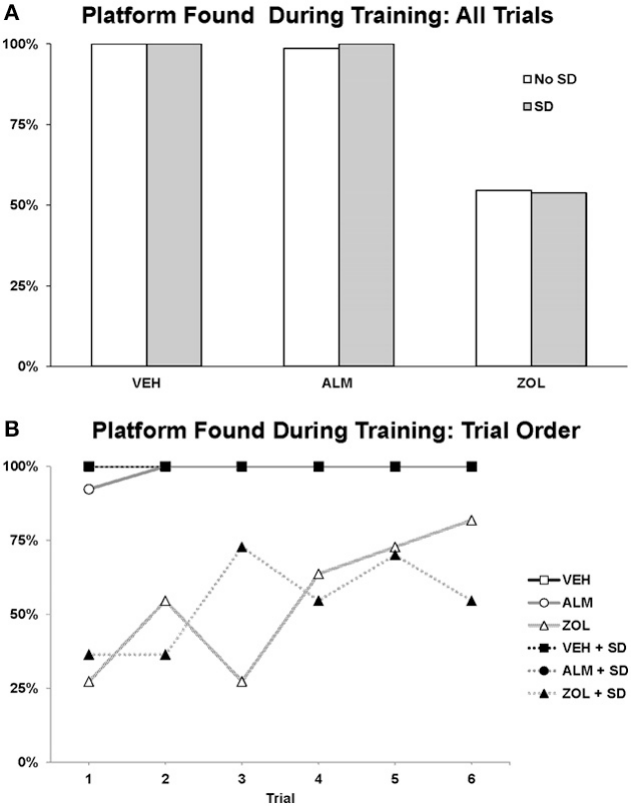
**Success rate in locating the platform during the training trials in the spatial working memory task**. The platform was cued during these training trials by a flag. **(A)** The percentage of times the platform was found across all 6 training trials. **(B)** The percentage of times the platform was found trial by trial. Note that the ZOL rats tended to progressively improve across trials. In each trial, an individual rat either found or didn't find the platform; thus, there is no variation to represent as error bars in the graphs.

The goal for the SWM task was to locate the platform. VEH- and ALM-treated rats found the platform the majority of the time in both SD and undisturbed conditions (83.3–100% for VEH and 79.2–87.5% for ALM; Figure 6). Conversely, ZOL-treated rats failed to find the platform most of the time (22.7–50.0% success rate). Interestingly, ZOL-treated rats also often failed to find the cued platform during the training phase of each pair of trials (Figure 7). The ZOL-treated rats in the baseline group found the cued platform 54.4% of the time while the SD ZOL-treated group were successful 53.8% of the time as compared to 98.6% for ALM-treated rats in the baseline group and 100% following SD and 100% of the time for all VEH-treated rats. A trend toward improved performance was observed with progressive trials in the ZOL-treated rats.

## Discussion

Though differing in the latency to induce sleep at the doses tested, ALM, and ZOL were equally effective at promoting sleep during the 90 min period prior to performance testing and both compounds significantly increased sleep compared to VEH. ALM-treated rats were indistinguishable from VEH-treated rats in their performance of both the SRM and SWM tasks. In contrast, ZOL caused significant impairments in both tasks. Specifically, in the SRM task, ZOL increased the latency to, the duration in, and the frequency of entering the target zone. In the SWM task, ZOL increased the time to find the platform, decreased the swim velocity and decreased the success rate in finding the platform. These results support the hypothesis that dual Hcrt receptor antagonism effectively promotes sleep without the functional impairments observed following GABA_A_ receptor modulation.

An alternative explanation of the results obtained is that ZOL-treated rats were not motivated to perform the tasks rather than having memory/cognitive deficits. ZOL-treated rats had decreased distance traveled during the SRM task and decreased velocity during the SWM task, which could indicate a lack of motivation to escape the WM. Further, the lower success rate in finding the cued platform during the training trials for the SWM task could be interpreted as an absence of motivation to escape. However, ZOL rats did not simply float in the WM; they swam continuously, primarily circling the perimeter of the WM. As mentioned above, this swim pattern is typical of an untrained rat during its first exposure to the WM. Although not measured in this study, it is possible that the decreased distance traveled during the SRM task and decreased velocity during the SWM task are due to motor deficits produced by ZOL. This hypothesis is supported by previous studies that found prominent motor effects following ZOL administration (Depoortere et al., 1986; Steiner et al., 2011; Milic et al., 2012).

The SD protocol in these studies was included to assess whether moderate increases in sleep drive would exacerbate any cognitive deficits found following ALM or ZOL administration and also produce deficits in VEH-treated rats. While the primary active period of nocturnal rodents such as the rat is during the dark phase, rats still sleep approximately 30% of the time during this period and increasing wake duration during the dark period should create a mild sleep deficit (see Figure 2). Therefore, a portion of our experimental protocol involved SD during the 6 h of the dark period just prior to drug administration at ZT18. Although we did not find significant effects of SD vs. non-SD within any of the 3 dosing conditions, these results are likely due to the fact that we allowed the rats to sleep after drug administration until water maze testing began. This undisturbed period lasted only 60–90 min but provided an opportunity for the experimental subjects to recover from this mild sleep deprivation. If the SD were continued until testing, increased memory deficits might have been observed. Further studies are needed to determine whether this is indeed to case.

ZOL is a widely prescribed hypnotic medication that can be well-tolerated when taken as directed (Greenblatt and Roth, 2012). However, numerous adverse effects associated with ZOL usage have been reported including driving impairment (Verster et al., 2006; Gunja, 2013), effects on balance and postural tone (Zammit et al., 2008), interference with memory consolidation (Balkin et al., 1992; Wesensten et al., 1996, 2005; Mintzer and Griffiths, 1999; Morgan et al., 2010) and increased incidence of complex behaviors during sleep (Hoever et al., 2010). Some studies investigated the effects of daytime administration of ZOL and tested psychomotor function upon arousal from naps (Wesensten et al., 2005; Storm et al., 2007), a protocol which our experiments closely mimic. In these studies, ZOL or melatonin was administered at either 10:00 or 13:00. Following a 1.5–2 h nap opportunity, subjects were awakened and required to perform a series of psychomotor and cognitive tests. Significant performance decrements were observed following ZOL in cognitive, vigilance and memory tasks while little to no decrements were found following melatonin. The results of ZOL administration on rat cognitive performance in the current study correlate well with these deficits found in humans.

In contrast, the high level of performance following ALM in both of our memory tasks suggests a high degree of safety at concentrations with hypnotic efficacy. Indeed, a recent study found no performance decrements in a variant of the WM SRM task at three-fold the concentration of ALM that we used (Dietrich and Jenck, 2010). Furthermore, another recent study found no effect of ALM at 300 mg/kg on motor function (Steiner et al., 2011). In humans, however, psychometric test battery assessment of the effect of ALM administered in the daytime found reductions in vigilance, alertness, and visuomotor and motor coordination at dose concentrations of 400–1000 mg (Hoever et al., 2010, 2012a). Notably, 400 mg ALM is within the therapeutic dose range required to improve sleep in patients with primary insomnia (Hoever et al., 2012b). Therefore, performance deficits following ALM occur within the range of hypnotic efficacy in humans. In one report, pharmacokinetic/pharmacodynamic modeling suggests that doses of 500 mg ALM and 10 mg ZOL are equivalent with respect to subjectively assessed alertness (Hoever et al., 2010). Since we find hypnotic efficacy to be achieved at roughly similar dose concentrations, there may be species differences in pharmacokinetic/pharmacodynamics of ALM and/or ZOL. While not uncommon, this makes direct translational interpretations of the present data more difficult. Regardless, in both rodents and humans, ALM appears to have a significantly better safety profile than ZOL with regards to cognitive/memory domains.

## Conclusion

ALM and ZOL are effective hypnotics in multiple mammalian species (Brisbare-Roch et al., 2007; Hoever et al., 2010, 2012a,b; Morairty et al., 2012). They act through entirely different mechanisms of action, and their effects on cognition, psychomotor vigilance and memory are in stark contrast to one another. We found that at equipotent hypnotic concentrations, ZOL impaired SRM and SWM but ALM did not. These results support the hypothesis that antagonism of the Hcrt system can provide hypnotic efficacy without the impairments found by inducing sleep through GABA_A_ modulation.

### Conflict of interest statement

The authors declare that the research was conducted in the absence of any commercial or financial relationships that could be construed as a potential conflict of interest.

## References

[B1] Balkin T. J. O'Donnell V. M. Wesensten N. McCann U. Belenky G. (1992). Comparison of the daytime sleep and performance effects of zolpidem versus triazolam. Psychopharmacology 107, 83–88 10.1007/BF02244970 1589566

[B2] Belenky G. Wesensten N. J. Thorne D. R. Thomas M. L. Sing H. C. Redmond D. P. (2003). Patterns of performance degradation and restoration during sleep restriction and subsequent recovery: a sleep dose-response study. J. Sleep Res. 12, 1–12 10.1046/j.1365-2869.2003.00337.x 12603781

[B3] Betschart C. Hintermann S. Behnke D. Cotesta S. Fendt M. Gee C. E. (2013). Identification of a novel series of orexin receptor antagonists with a distinct effect on sleep architecture for the treatment of insomnia. J. Med. Chem. 56, 7590–7607 10.1021/jm4007627 23964859

[B4] Brisbare-Roch C. Dingemanse J. Koberstein R. Hoever P. Aissaoui H. Flores S. (2007). Promotion of sleep by targeting the orexin system in rats, dogs and humans. Nat. Med. 13, 150–155 10.1038/nm1544 17259994

[B5] Coleman P. J. Schreier J. D. Cox C. D. Breslin M. J. Whitman D. B. Bogusky M. J. (2012). Discovery of [(2R,5R)-5-{[(5-fluoropyridin-2-yl)oxy]methyl}-2-methylpiperidin-1-yl][5-methyl-2 -(pyrimidin-2-yl)phenyl]methanone (MK-6096): a dual orexin receptor antagonist with potent sleep-promoting properties. ChemMedChem 7, 415–424, 337. 10.1002/cmdc.201200025 22307992

[B6] de Lecea L. (2012). Hypocretins and the neurobiology of sleep-wake mechanisms. Prog. Brain Res. 198, 15–24 10.1016/B978-0-444-59489-1.00003-3 22813967 PMC5049885

[B7] Depoortere H. Zivkovic B. Lloyd K. G. Sanger D. J. Perrault G. Langer S. Z. Bartholini G. (1986). Zolpidem, a novel nonbenzodiazepine hypnotic. I. Neuropharmacological and behavioral effects. J. Pharmacol. Exp. Ther. 237, 649–658 2871178

[B8] Dietrich H. Jenck F. (2010). Intact learning and memory in rats following treatment with the dual orexin receptor antagonist almorexant. Psychopharmacology 212, 145–154 10.1007/s00213-010-1933-5 20631993 PMC2937139

[B9] Dolder C. R. Nelson M. H. (2008). Hypnosedative-induced complex behaviours: incidence, mechanisms and management. CNS Drugs 22, 1021–1036 10.2165/0023210-200822120-00005 18998740

[B10] Dugovic C. Shelton J. E. Aluisio L. E. Fraser I. C. Jiang X. Sutton S. W. (2009). Blockade of orexin-1 receptors attenuates orexin-2 receptor antagonism-induced sleep promotion in the rat. J. Pharmacol. Exp. Ther. 330, 142–151 10.1124/jpet.109.152009 19363060

[B11] Greenblatt D. J. Roth T. (2012). Zolpidem for insomnia. Expert Opin. Pharmacother. 13, 879–893 10.1517/14656566.2012.667074 22424586

[B12] Gunja N. (2013). In the Zzz zone: the effects of z-drugs on human performance and driving. J. Med. Toxicol. 9, 163–171 10.1007/s13181-013-0294-y 23456542 PMC3657033

[B13] Herring W. J. Snyder E. Budd K. Hutzelmann J. Snavely D. Liu K. (2012). Orexin receptor antagonism for treatment of insomnia: a randomized clinical trial of suvorexant. Neurology 79, 2265–2274 10.1212/WNL.0b013e31827688ee 23197752

[B14] Hoever P. de Haas S. Winkler J. Schoemaker R. C. Chiossi E. van Gerven J. Dingemanse J. (2010). Orexin receptor antagonism, a new sleep-promoting paradigm: an ascending single-dose study with almorexant. Clin. Pharmacol. Ther. 87, 593–600 10.1038/clpt.2010.19 20376002

[B15] Hoever P. de Haas S. L. Dorffner G. Chiossi E. van Gerven J. M. Dingemanse J. (2012a). Orexin receptor antagonism: an ascending multiple-dose study with almorexant. J. Psychopharmacol. 26, 1071–1080 10.1177/0269881112448946 22695489

[B16] Hoever P. Dorffner G. Benes H. Penzel T. Danker-Hopfe H. Barbanoj M. J. (2012b). Orexin receptor antagonism, a new sleep-enabling paradigm: a proof-of-concept clinical trial. Clin. Pharmacol. Ther. 91, 975–985 10.1038/clpt.2011.370 22549286 PMC3370822

[B17] Huang M. P. Radadia K. Macone B. W. Auerbach S. H. Datta S. (2010). Effects of eszopiclone and zolpidem on sleep-wake behavior, anxiety-like behavior and contextual memory in rats. Behav. Brain Res. 210, 54–66 10.1016/j.bbr.2010.02.018 20153782 PMC2844486

[B18] Inutsuka A. Yamanaka A. (2013). The regulation of sleep and wakefulness by the hypothalamic neuropeptide orexin/hypocretin. Nagoya J. Med. Sci. 75, 29–36 23544265 PMC4345701

[B19] Lamond N. Jay S. M. Dorrian J. Ferguson S. A. Jones C. Dawson D. (2007). The dynamics of neurobehavioural recovery following sleep loss. J. Sleep Res. 16, 33–41 10.1111/j.1365-2869.2007.00574.x 17309761

[B20] Mai E. Buysse D. J. (2008). Insomnia: prevalence, impact, pathogenesis, differential diagnosis, and evaluation. Sleep Med. Clin. 3, 167–174 10.1016/j.jsmc.2008.02.001 19122760 PMC2504337

[B21] Makaron L. Moran C. A. Namjoshi O. Rallapalli S. Cook J. M. Rowlett J. K. (2013). Cognition-impairing effects of benzodiazepine-type drugs: role of GABAA receptor subtypes in an executive function task in rhesus monkeys. Pharmacol. Biochem. Behav. 104, 62–68 10.1016/j.pbb.2012.12.018 23290931 PMC3977599

[B22] Marcus J. N. Aschkenasi C. J. Lee C. E. Chemelli R. M. Saper C. B. Yanagisawa M. (2001). Differential expression of orexin receptors 1 and 2 in the rat brain. J. Comp. Neurol. 435, 6–25 10.1002/cne.1190 11370008

[B23] Mattila M. J. Vanakoski J. Kalska H. Seppala T. (1998). Effects of alcohol, zolpidem, and some other sedatives and hypnotics on human performance and memory. Pharmacol. Biochem. Behav. 59, 917–923 10.1016/S0091-3057(97)00506-6 9586849

[B24] Mieda M. Sakurai T. (2013). Orexin (hypocretin) receptor agonists and antagonists for treatment of sleep disorders. Rationale for development and current status. CNS Drugs 27, 83–90 10.1007/s40263-012-0036-8 23359095

[B25] Milic M. Divljakovic J. Rallapalli S. van Linn M. L. Timic T. Cook J. M. (2012). The role of alpha1 and alpha5 subunit-containing GABAA receptors in motor impairment induced by benzodiazepines in rats. Behav. Pharmacol. 23, 191–197 10.1097/FBP.0b013e3283512c85 22327019 PMC3296822

[B26] Mintzer M. Z. Griffiths R. R. (1999). Selective effects of zolpidem on human memory functions. J. Psychopharmacol. 13, 18–131 10.1177/026988119901300103 10221356

[B27] Morairty S. R. Hedley L. Flores J. Martin R. Kilduff T. S. (2008). Selective 5HT2A and 5HT6 receptor antagonists promote sleep in rats. Sleep 31, 34–44 18220076 10.1093/sleep/31.1.34 PMC2225549

[B28] Morairty S. R. Revel F. G. Malherbe P. Moreau J. L. Valladao D. Wettstein J. G. (2012). Dual hypocretin receptor antagonism is more effective for sleep promotion than antagonism of either receptor alone. PLoS ONE 7:e39131 10.1371/journal.pone.0039131 22768296 PMC3388080

[B29] Morgan P. T. Kehne J. H. Sprenger K. J. Malison R. T. (2010). Retrograde effects of triazolam and zolpidem on sleep-dependent motor learning in humans. J. Sleep Res. 19, 157–164 10.1111/j.1365-2869.2009.00757.x 19682231

[B30] NIH. (2005). National Institutes of Health State of the Science Conference statement on Manifestations and Management of Chronic Insomnia in Adults. Sleep 28, 1049–57 16268373 10.1093/sleep/28.9.1049

[B31] Otmani S. Demazieres A. Staner C. Jacob N. Nir T. Zisapel N. (2008). Effects of prolonged-release melatonin, zolpidem, and their combination on psychomotor functions, memory recall, and driving skills in healthy middle aged and elderly volunteers. Hum. Psychopharmacol. 23, 693–705 10.1002/hup.980 18763235

[B32] Peyron C. Tighe D. K. van den Pol A. N. de Lecea L. Heller H. C. Sutcliffe J. G. (1998). Neurons containing hypocretin (orexin) project to multiple neuronal systems. J. Neurosci. 18, 9996–10015 9822755 10.1523/JNEUROSCI.18-23-09996.1998 PMC6793310

[B33] Revel F. G. Moreau J. L. Gainetdinov R. R. Ferragud A. Velazquez-Sanchez C. Sotnikova T. D. (2012). Trace amine-associated receptor 1 partial agonism reveals novel paradigm for neuropsychiatric therapeutics. Biol. Psychiatry 72, 934–942 10.1016/j.biopsych.2012.05.014 22705041

[B34] Revel F. G. Moreau J. L. Pouzet B. Mory R. Bradaia A. Buchy D. (2013). A new perspective for schizophrenia: TAAR1 agonists reveal antipsychotic- and antidepressant-like activity, improve cognition and control body weight. Mol. Psychiatry 18, 543–556 10.1038/mp.2012.57 22641180

[B35] Roth T. (2007). Insomnia: definition, prevalence, etiology, and consequences. J. Clin. Sleep Med. 3, S7–S10 17824495 PMC1978319

[B36] Saper C. B. (2013). The neurobiology of sleep. Continuum (Minneap Minn) 19, 19–31 10.1212/01.CON.0000427215.07715.73 23385692

[B37] Soto P. L. Ator N. A. Rallapalli S. K. Biawat P. Clayton T. Cook J. M. (2013). Allosteric modulation of GABAA receptor subtypes: effects on visual recognition and visuospatial working memory in rhesus monkeys. Neuropsychopharmacology 38, 2315–2325 10.1038/npp.2013.137 23722241 PMC3773684

[B38] Steiner M. A. Lecourt H. Strasser D. S. Brisbare-Roch C. Jenck F. (2011). Differential effects of the dual orexin receptor antagonist almorexant and the GABA(A)-alpha1 receptor modulator zolpidem, alone or combined with ethanol, on motor performance in the rat. Neuropsychopharmacology 36, 848–856 10.1038/npp.2010.224 21150905 PMC3055732

[B39] Storm W. F. Eddy D. R. Welch C. B. Hickey P. A. Fischer J. Cardenas R. (2007). Cognitive performance following premature awakening from zolpidem or melatonin induced daytime sleep. Aviat. Space Environ. Med. 78, 10–20 17225476

[B40] Uslaner J. M. Tye S. J. Eddins D. M. Wang X. Fox S. V. Savitz A. T. (2013). Orexin receptor antagonists differ from standard sleep drugs by promoting sleep at doses that do not disrupt cognition. Sci. Transl. Med. 5, 179ra44 10.1126/scitranslmed.3005213 23552372

[B41] Vanover K. E. Edgar D. M. Seidel W. F. Hogenkamp D. J. Fick D. B. Lan N. C. (1999). Response-rate suppression in operant paradigm as predictor of soporific potency in rats and identification of three novel sedative-hypnotic neuroactive steroids. J. Pharmacol. Exp. Ther. 291, 1317–1323 10565857

[B42] Verster J. C. Veldhuijzen D. S. Patat A. Olivier B. Volkerts E. R. (2006). Hypnotics and driving safety: meta-analyses of randomized controlled trials applying the on-the-road driving test. Curr. Drug Saf. 1, 63–71 10.2174/157488606775252674 18690916

[B43] Verster J. C. Volkerts E. R. Schreuder A. H. Eijken E. J. van Heuckelum J. H. Veldhuijzen D. S. (2002). Residual effects of middle-of-the-night administration of zaleplon and zolpidem on driving ability, memory functions, and psychomotor performance. J. Clin. Psychopharmacol. 22, 576–583 10.1097/00004714-200212000-00007 12454557

[B44] Ward C. P. McCarley R. W. Strecker R. E. (2009). Experimental sleep fragmentation impairs spatial reference but not working memory in Fischer/Brown Norway rats. J. Sleep Res. 18, 238–244 10.1111/j.1365-2869.2008.00714.x 19645967 PMC2721795

[B45] Wenk G. L. (2004). Assessment of spatial memory using the radial arm maze and Morris water maze. Curr. Protoc. Neurosci. Chapter 8, Unit 8.5A. 10.1002/0471142301.ns0805as26 18428607

[B46] Wesensten N. J. Balkin T. J. Belenky G. (1999). Does sleep fragmentation impact recuperation? A review and reanalysis. J. Sleep Res. 8, 237–245 10.1046/j.1365-2869.1999.00161.x 10646163

[B47] Wesensten N. J. Balkin T. J. Belenky G. L. (1996). Effects of daytime administration of zolpidem and triazolam on performance. Aviat. Space Environ. Med. 67, 115–120 8834935

[B48] Wesensten N. J. Balkin T. J. Reichardt R. M. Kautz M. A. Saviolakis G. A. Belenky G. (2005). Daytime sleep and performance following a zolpidem and melatonin cocktail. Sleep 28, 93–103 15700725 10.1093/sleep/28.1.93

[B49] Whitman D. B. Cox C. D. Breslin M. J. Brashear K. M. Schreier J. D. Bogusky M. J. (2009). Discovery of a potent, CNS-penetrant orexin receptor antagonist based on an n,n-disubstituted-1,4-diazepane scaffold that promotes sleep in rats. ChemMedChem 4, 1069–1074 10.1002/cmdc.200900069 19418500

[B50] Winrow C. J. Gotter A. L. Cox C. D. Tannenbaum P. L. Garson S. L. Doran S. M. (2012). Pharmacological characterization of MK-6096 - a dual orexin receptor antagonist for insomnia. Neuropharmacology 62, 978–987 10.1016/j.neuropharm.2011.10.003 22019562

[B51] Zammit G. Wang-Weigand S. Peng X. (2008). Use of computerized dynamic posturography to assess balance in older adults after nighttime awakenings using zolpidem as a reference. BMC Geriatr. 8:15 10.1186/1471-2318-8-15 18627623 PMC2492850

[B52] Zanin K. A. Patti C. L. Sanday L. Fernandes-Santos L. Oliveira L. C. Poyares D. (2013). Effects of zolpidem on sedation, anxiety, and memory in the plus-maze discriminative avoidance task. Psychopharmacology 226, 459–474 10.1007/s00213-012-2756-3 22729271

